# Performance evaluation of the PennPET explorer with expanded axial coverage

**DOI:** 10.1088/1361-6560/acc722

**Published:** 2023-04-19

**Authors:** Bing Dai, Margaret E Daube-Witherspoon, Stephen McDonald, Matthew E Werner, Michael J Parma, Michael J Geagan, Varsha Viswanath, Joel S Karp

**Affiliations:** 1 Department of Radiology, University of Pennsylvania, Philadelphia, United States of America

**Keywords:** total-body PET, NEMA performance, axial detector gaps

## Abstract

*Objective.* This work evaluated the updated PennPET Explorer total-body (TB) PET scanner, which was extended to 6 rings with updated readout firmware to achieve a 142 cm axial field of view (AFOV) without 7.6 cm inter-ring axial gaps. *Approach.* National Electrical Manufacturers Association (NEMA) NU 2-2018 measurements were performed with modifications including longer phantoms for sensitivity and count-rate measurements and additional positions for spatial resolution and image quality. A long uniform phantom and the clinical trials network (CTN) phantom were also used. *Main results.* The total sensitivity increased to 140 kcps MBq^−1^ for a 70 cm line, a gain of 1.8x compared to the same system with axial gaps; an additional 47% increase in total counts was observed with a 142 cm line at the same activity per cm. The noise equivalent count rate (NECR) increased by 1.8x without axial gaps. The peak NECR is 1550 kcps at 25 kBq cc^−1^ for a 140 cm phantom; due to increased randoms, the NECR is lower than with a 70 cm phantom, for which NECR is 2156 kcps cc^−1^ at 25 kBq cc^−1^ and continues increasing. The time-of-flight resolution is 250 ps, increasing by <10 ps at the highest activity. The axial spatial resolution degrades by 0.6 mm near the center of the AFOV, compared to 4 mm resolution near the end. The NEMA image quality phantom showed consistent contrast recovery throughout the AFOV. A long uniform phantom demonstrated axial uniformity of uptake and noise, and the CTN phantom demonstrated quantitative accuracy for both ^18^F and ^89^Zr. *Significance*. The performance evaluation of the updated PennPET Explorer demonstrates significant gains compared to conventional scanners and shows where the current NEMA standard needs to be updated for TB-PET systems. The comparisons of systems with and without inter-ring gaps demonstrate the performance trade-offs of a more cost-effective TB-PET system with incomplete detector coverage.

## Introduction

Total-body (TB) PET systems (Karp *et al* 2020, Spencer *et al* 2021, Prenosil *et al* 2022) have two major advantages over conventional scanners with standard axial field of view (AFOV): high sensitivity and the capability to image dynamic processes in multiple organs of the body simultaneously (Badawi *et al* 2021).

The PennPET Explorer, a TB PET system, was developed as a scalable, long AFOV scanner. Its performance has previously been characterized in two interim geometries: a 3-ring configuration with 64 cm AFOV and 5-ring configuration with 112 cm AFOV, both with inter-ring axial gaps (Karp *et al* 2020, Viswanath *et al* 2020). The PennPET Explorer with 3-ring (64 cm AFOV) and 5-ring (112 cm AFOV) configurations demonstrated excellent quality and quantitative accuracy for human imaging across the AFOV (Pantel *et al* 2020), despite incomplete detector coverage due to the factory readout firmware that could read only 5 of the 7 rows of detectors/ring. The PennPET Explorer was recently extended to 6 rings, and the data acquisition firmware was concurrently updated from 5-row to 7-row readout, thus activating all detectors and achieving a 142 cm AFOV without gaps between the rings. The upgrades provide the opportunity to measure the performance of a long AFOV system in its completed configuration, as well as to quantify the performance trade-offs associated with large gaps between rings.

**Figure 1.**
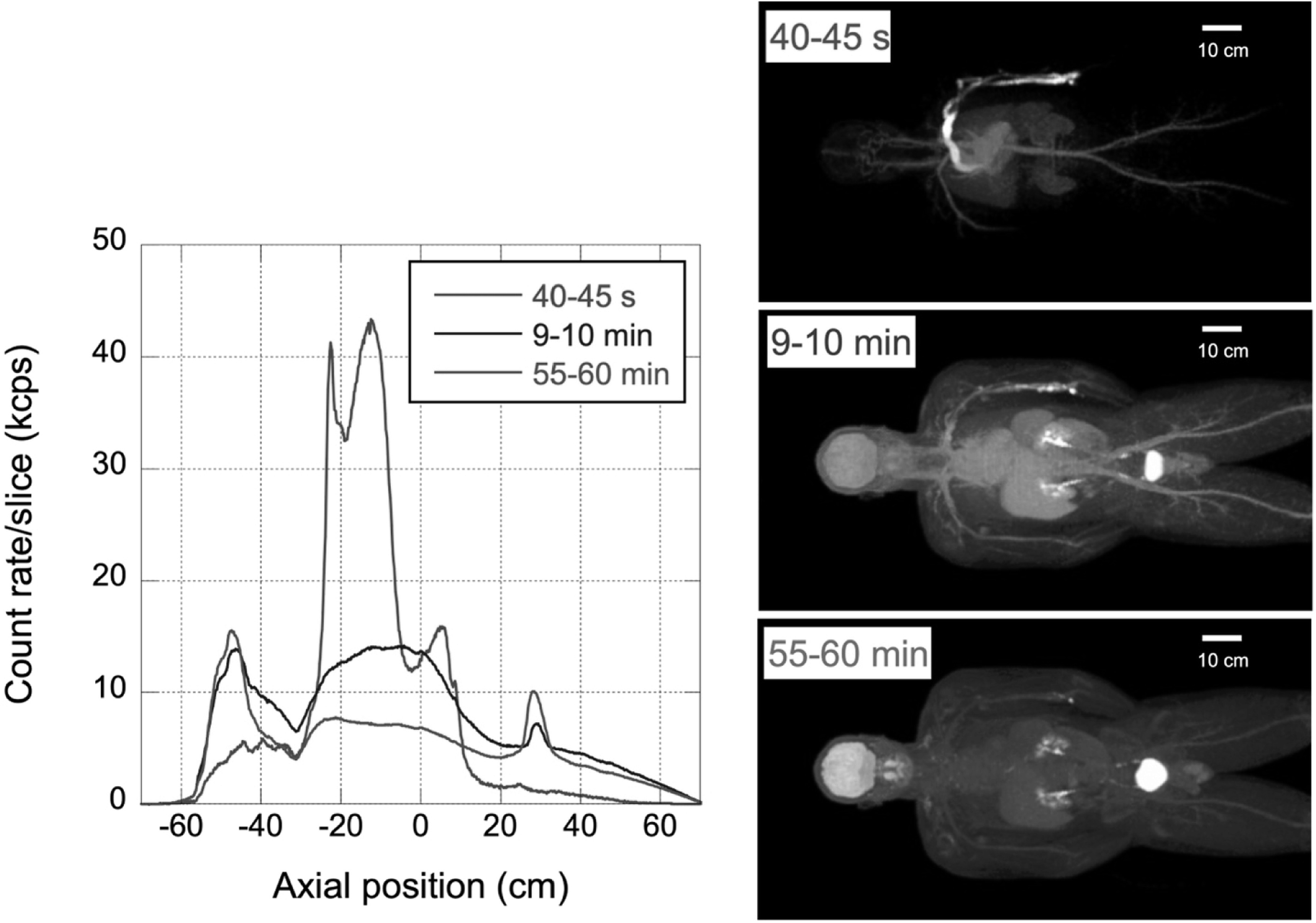
Representative maximum intensity projection (MIP) images and axial count rate (trues + scatter) profiles on the PennPET Explorer with 142 cm AFOV at three time points in a dynamic [^18^F]-fluorodeoxyglucose (FDG) study (349 MBq injection) for a male patient of average height and weight (174 cm and 88 kg).

The National Electrical Manufacturers Association (NEMA) standard, developed for PET scanners with a maximum length of 65 cm (NEMA NU 2-2018, 2018), was adequate to characterize the PennPET Explorer scanner in its original configuration with a 64 cm AFOV (3 rings), but some tests included in the standard cannot capture the full benefit of the PennPET Explorer in its current configuration (142 cm) or other long AFOV systems, including the United Imaging uEXPLORER (194 cm) and the Siemens Vision Quadra (106 cm) scanners. Figure 1 shows representative images and axial count profiles on the PennPET Explorer at three time points in a dynamic patient study to illustrate the challenges of characterizing the performance of a TB-PET system using the current standard. In particular, the sensitivity and count rate measurements both rely on phantoms of 70 cm length and therefore can underestimate the performance gains of long AFOV systems. The sequence of images in figure 1 shows the varying count rate distribution over time, although since the total dose is in the FOV for the 1 h scan, the summed (Trues + Scatter) count rate is approximately constant (except for decay). These distributions show that the majority of activity for an average size adult is at least 100 cm in axial dimension and suggest that a phantom longer than 70 cm will better characterize the performance of a TB-PET scanner and will be more clinically relevant in helping to guide the development of new scan protocols.

In this work, the design of the PennPET Explorer is first described, followed by the data flow from the perspective of the data acquisition firmware. The upgrade to the firmware to enable all detectors is then explained. NEMA NU-2 2018 measurements with modifications to the lengths and positions of phantoms, scan durations, etc., were performed on the 6-ring system both with and without axial gaps, along with additional phantom studies and tests. The system performance in terms of sensitivity, image uniformity, count rate, timing resolution, spatial resolution, and image quality are presented and discussed.

## Materials and methods

### Design of the PennPET Explorer

The PennPET Explorer is a modular, scalable, and programmable TB PET system in terms of hardware, firmware, and software. It has been operated for human imaging in configurations ranging from 3 to 6 rings.

Each ring is identical, measuring 76.4 cm in diameter and 22.9 cm axially. There is a 1.1 cm physical gap between rings. Each ring comprises 18 identical modules. Each module has 28 detector tiles in a 4 × 7 array; each row is 3.26 cm in the axial direction. In each tile, a 64-channel digital silicon photomultiplier (SiPM) developed by Philips Digital Photon Counting (PDPC) is coupled to an 8 × 8 array of 3.86 × 3.86 × 19 mm^3^ lutetium-yttrium oxyorthosilicate (LYSO) scintillation crystals. The fully digital SiPM sensor tile has 4 × 4 individual dies, each generating an independent time stamp, and each die has 2 × 2 pixels, allowing a 1:1 sensor-crystal coupling that ensures superior timing readout and avoids light pile-up so that the detectors have very small deadtime. The scanner is water-cooled to 10 °C to minimize noise and deadtime of the SiPM sensor and allow for using the first photon trigger level to optimize the timing resolution (Degenhardt *et al* 2009, 2012, Frach *et al* 2009). The room temperature is kept around 20 °C. Further details regarding the scanner design were described in Karp *et al* (2020).

The firmware performs critical control, monitoring, and data manipulation functions for the system. Specifically, the basic data processing and communication blocks on the tiles, sensor boards, main boards, and coincidence detection unit (CDU) are designed on field programmable gate arrays (FPGAs) to cope with the high-speed, low latency data streaming required by the PET system while reserving enough resources for fast digital signal processing and communication through the entire system stack. One distinct advantage of the FPGA design is its re-programmability that allowed us to update the readout firmware.

The processing software is also flexible. It permits using a subset of the 6 rings, as appropriate for the radiotracer or study (e.g. pediatric or brain studies), to allow for efficient data processing.

### Data acquisition

The data acquisition for each ring is based on that of the Philips Vereos PET-CT, which was the first commercial PET/CT introduced with SiPM-based detectors (Miller *et al* 2015, Rausch *et al* 2019) and provided significantly improved TOF resolution compared to the competing PMT-based systems from other vendors. However, Philips made a decision during the development of the Vereos PET/CT to limit the system to 16.4 cm AFOV (5 detector rows); thus, the factory firmware, more specifically, the factory sorter/merger on the main board could only handle 5 channels (or 5 detector rows) of input data, even though the electronics were designed for 7 detector rows. In the development of the multi-ring PennPET Explorer scanner it was decided to maximize the AFOV by spacing the rings apart based on 7 rows (22.9 cm axial length), even though only 5 rows were active. Thus, before the firmware was modified, the PennPET Explorer operated with *7.6* cm axial gaps between rings, including a *1.1* cm physical gap between rings. To clearly distinguish the scanner with and without inactive detector rows, we use ‘with axial gaps’ to denote 7.6 cm axial gaps (including the physical gap), and ‘without axial gaps’ to denote only the 1.1 cm physical gaps in the rest of the paper.

**Figure 2.**
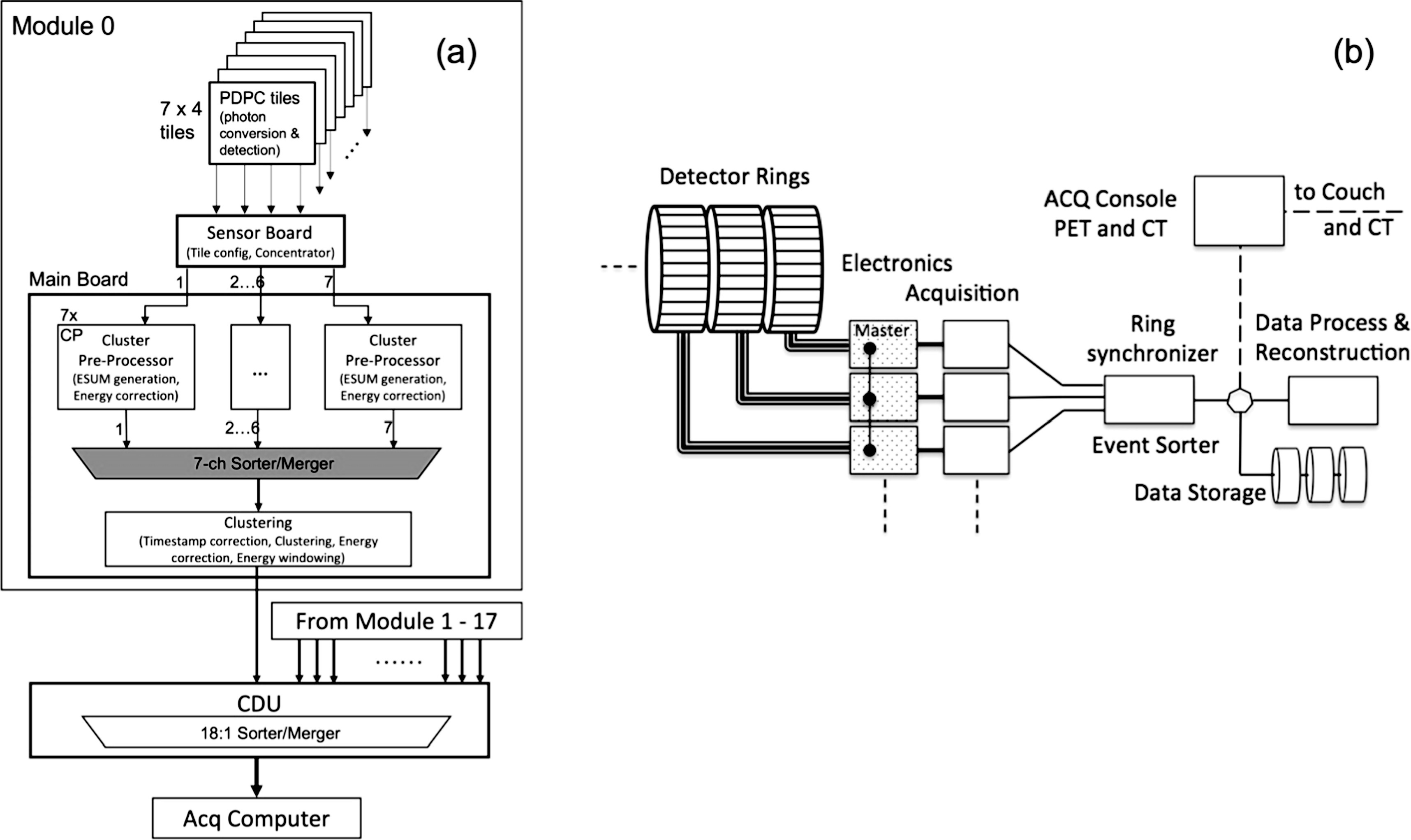
(a) Data flow of a single detector module in the PennPET Explorer. (b) Schematic of multi-ring data acquisition.

As illustrated in figure 2(a), for each module the 28 tiles detect and convert photons to singles events with energy (in terms of photon counts) and timestamps. The events from the die sensor are processed by the tile FPGA in 320 *μ*s time frames.

The sensor board connects to the tiles. Each FPGA on the sensor board interfaces with 4 tiles in a row. It configures the tiles with instructions required for operation, including trigger levels and inhibit maps (Degenhardt *et al* 2009). In addition, it works as a concentrator to merge the data streams of 4 tiles to one stream. Therefore, the sensor board sends 7 channels of data to the main board for further processing.

The main board performs some of the most important functions for the system. It is composed of three major functional blocks. (1) Cluster pre-processor: There are 7 of these to cope with the 7 input channels. It converts the PDPC time frames from the tiles with multiple events per packet into small packets with only one event per die, including changing PDPC addressing to a linear address scheme where each crystal (or pixel) has a linear tangential (*x*) and axial (*z*) address, selecting the timestamp for each crystal, and summing up photon counts of all four pixels of a die. (2) Sorter/Merger: It sorts all channels of input data by timestamps and merges them to one stream. (3) Clustering: It performs timestamp correction, clustering, post-clustering energy correction, and energy windowing for the merged stream and sends the data to the CDU.

All 18 modules in a ring are identical and work independently. They connect to the same CDU, which sorts/merges 18 channels of data to one data stream and sends it to the acquisition computer for the ring. Each ring has its own acquisition computer, and the rings operate independently, although they are synchronized through a common clock signal provided by ring 1. Note that the CDU was designed to determine coincidence pairs of events in a single ring of 18 modules, but since data are collected from multiple rings, the CDU is only used to send the data stream of ‘singles’ events to the acquisition computers, each with 3T-bytes of storage. Coincidence events are subsequently determined, in software in real-time with data acquisition, from all possible pairs of detectors using a multiple-window coincidence sorting policy that accepts all combinations of 2-detector pairs of singles events.

### Firmware upgrade

While the FPGA is reprogrammable, its limited on-board resources did not allow a straightforward addition of two channels to the original 5-channel design. In fact, the firmware architecture of the sorter/merger on the main board had to be redesigned in order to read out all 7 rows of data by reducing the number of layers in the sorting chain thus reducing the consumption of on-board resources. In addition, a new FIFO was adopted to provide better control of data streaming, e.g. backpressure that allows the downstream to slow down the previous stage when the upstream data rate is too high for the downstream to process without losses.

With the new readout firmware, the 6-ring PennPET Explorer is fully populated, achieving 142 cm AFOV without axial gaps. This was the original goal of the design, although the scanner operated in both 3-ring and 5-ring configurations with inter-ring gaps (Karp *et al* 2020, Viswanath *et al* 2020). The subsequent sections will demonstrate the performance trade-offs with and without axial gaps, and the comparison can be used to consider trade-offs for future designs with sparse detector coverage (Yamaya *et al* 2009, Zein *et al* 2021), whether for TB-PET or for PET systems with conventional AFOV.

### Performance characterization

As noted earlier, the NEMA NU-2 metrics may not adequately reflect the performance differences of TB-PET systems compared to those with standard AFOV. The sensitivity and count rate benefits are not captured by the shorter phantoms prescribed. In addition, the wide variation of sensitivity across the AFOV calls for performance measurements at locations other than the axial center. Therefore, both standard and modified NEMA measurements as well as additional phantoms were performed on the PennPET Explorer with both factory firmware (5-row readout) and the updated firmware (7-row readout) to evaluate the trade-offs between the system performance with/without axial gaps. For all the measurements reported in this work, an energy window of 450–630 keV and a coincidence window (*τ*) of 4.5 ns were used.

#### Sensitivity

The standard 70 cm line source prescribed by NEMA 2-2018 for sensitivity measurement cannot fully measure the axial sensitivity profile across the entire system and, therefore, does not reflect the measured events seen clinically in TB-PET systems (where the activity distribution often extends beyond 70 cm). As such, in addition to the 70 cm line source, the sensitivity of the 6-ring PennPET Explorer was also measured with a line source equal to the scanner length to characterize the full sensitivity of the system. The 70 cm line was filled with an initial activity ∼21 MBq of ^18^F and suspended at the center of the scanner. A 142 cm line that extended the full AFOV of the system was filled with ∼34 MBq. In both cases, following the NEMA 2-2018 protocol, attenuation-free sensitivity was extrapolated by using a set of five concentric aluminum attenuating sleeves with lengths matching the length of the line source. Axial sensitivity profiles were created by binning the list-mode data into histo-images with slice thickness of 2 mm (Matej *et al* 2009) using the TOF information to place the events at their most likely axial position. For the 5-ring and 6-ring configurations with gaps where the sensitivity for a 70 cm line was not measured, the total sensitivity for a 70 cm line was obtained from the 142 cm axial sensitivity profile by summing only the central 70 cm of the axial sensitivity profile and multiplying by 142/70 to consider only the activity in the 70 cm length. This extrapolation was verified on the 6-ring scanner without gaps; the 70 cm result extrapolated from the 142 cm measurement agreed to within 3% of the 70 cm measurement.

#### Image uniformity

Image uniformity has not been part of the NEMA NU-2 standard, although we believe that axial uniformity is an important metric to consider for long AFOV systems because of their large axial variations in sensitivity. We chose a 10 cm diameter, 190 cm long pipe to keep a reasonable weight for a long phantom to assess the uniformity of image noise and quantitative accuracy throughout the AFOV. This phantom is also used to define the calibration factor for the system. It was uniformly filled with 120 MBq (8.4 kBq cc^−1^) of ^18^F, comparable to the activity concentration seen for patient studies on the PennPET Explorer, and imaged for 15 min. Images were reconstructed using list-mode TOF ordered subsets expectation maximization (LM-TOF-OSEM; 25 subsets, 5 iterations) into 2 × 2 × 2 mm^3^ voxels (Popescu *et al* 2004). The same parameters were used for all the reconstructions reported in this work unless otherwise specified. The TOF-enhanced single scatter simulation was used for scatter estimation (Werner *et al* 2006).

Axial uniformity was calculated on the reconstructed images by placing an 80 mm diameter circular region of interest (ROI) on every image slice and measuring the mean standardized uptake value (SUV) in the ROI. Axial image noise was characterized using the image roughness, calculated as the ratio of the standard deviation (SD) of SUV within the ROI to the mean SUV for each slice.

#### Count rate, accuracy and time-of-flight resolution

The count rate performance was first measured with the standard 20 × 70 cm NEMA phantom—a 70 cm long line source placed 4.5 cm off-center in a 20 × 70 cm polyethylene phantom. As injected activity does not leave the AFOV of a TB-PET system during a dynamic study (except through physical decay), a 70 cm phantom, shorter than the activity distribution in clinical studies, can underestimate randoms and overestimate the noise-equivalent count (NEC) rate seen with human imaging. For this reason, the measurement was repeated with two 70 cm phantoms placed back-to-back to form a 20 × 140 cm distribution.

The single 20 × 70 cm phantom was imaged on the 6-ring PennPET Explorer only in its current configuration, without axial gaps. The 70 cm line source inside the phantom was filled with ∼1000 MBq of ^18^F and decayed for 10 h, resulting in activity concentrations of ∼1–45 kBq cc^−1^. The double 20 × 140 cm phantom was imaged on the 6-ring system both before and after the firmware update, thus, with and without axial gaps. For the system configuration with gaps, the 140 cm line source was filled with ∼1800 MBq of ^18^F and decayed for 11.7 h, resulting in activity concentrations of ∼0.5–40 kBq cc^−1^. For the current system configuration without axial gaps, the initial injection activity was reduced to ∼1380 MBq (30 kBq cc^−1^) to avoid a very high singles rate that would overload the data acquisition hardware due to the increased sensitivity of the complete system.

The accuracy of the correction for dead time losses was calculated from the reconstructed images of the 20 × 70 cm phantom. Data were reconstructed using LM-TOF-OSEM (2 × 2 × 2 mm^3^ voxels) and corrected for decay. No deadtime correction was applied during reconstruction. A 16 cm diameter cylindrical volume of interest (VOI) was drawn in the reconstructed image for each frame. The true rate was calculated by averaging the last 5 data points. The loss of accuracy (i.e. deadtime) was calculated as the percent error between the VOI and true rates.

The time-of-flight (TOF) resolution was calculated from both single and double count rate phantom measurements on the 6-ring scanner without axial gaps following the NEMA protocol. The timing resolution of single rings was obtained from daily quality control (QC) measurements with a ^22^Na (8.1 MBq) point source.

#### Spatial resolution

Spatial resolution was measured with a 0.25 mm diameter ^22^Na point source (75 kBq) encased in a 1 cm^3^ plastic cube. Measurements were performed at five radial positions (1, 5, 10, 15, 20 cm) and six axial positions (0, 12, 24, 36, 48, 60 cm from the center), more than the NEMA standard requires, to more completely characterize spatial resolution throughout the field-of-view for the TB-PET system. For axial positions, the point source was placed between rings and in the centers of the 4th, 5th and 6th rings corresponding to 1-ring (smallest axial acceptance angle, ±16.7°), 3-ring (±43°), and 6-ring (largest acceptance angle, ±62°) configurations, to characterize the dependence of axial resolution on acceptance angle.

Although prescribed by NEMA, analytic reconstruction (filtered back-projection, FBP) is subject to point spread function (PSF) distortions from rebinning or undersampling errors that are not observed in images reconstructed with the (iterative) clinical algorithm. This is shown in table 1 where radial, tangential, and axial resolutions are reported at radial positions of 1 and 20 cm for a single ring of the PennPET Explorer with analytic and iterative reconstruction algorithms. In addition to the usual degradation of radial resolution with increased radial offset caused by parallax errors, tangential and axial resolutions also appear to degrade at large radial offsets when analytic reconstruction (3DFRP (Matej and Lewitt 2001)) is used, whereas the PSF distortions and anomalous results are eliminated with iterative reconstruction. The errors are exacerbated for the large axial angles and large sinogram sizes of a long AFOV system where memory limitations can restrict the accuracy of rebinning and sampling. In addition, analytic reconstruction requires complete sampling, which was not possible for the system configuration with axial gaps. Therefore, LM-TOF-OSEM iterative reconstruction with clinical parameters was applied, except that 1 mm voxels were used to avoid undersampling in defining the full-width at half-maximum (FWHM) and full-width at tenth-maximum (FWTM). Note that our iterative algorithm includes spherical image basis functions optimized in size and grid spacing for the spatial resolution and noise characteristics of the imager (Matej and Lewitt 1996), as opposed to cubic voxels. These spherical image basis functions eliminate the over-convergence of OSEM (which can lead to overestimated spatial resolution performance (Gong *et al* 2016) when reconstructing a point source in air; they also suppress image noise while preserving signal, so no postfiltering is needed. The spatial resolution was calculated by fitting the three maximal points to a parabolic function and linearly interpolating to calculate the FWHM and FWTM. Radial and tangential resolutions were averaged over all axial source positions at a given radial position, and axial resolutions were averaged over all transverse positions. Uncertainties were calculated as the standard deviation (SD) over the different source positions.

**Table 1.** Spatial resolution achieved for a single ring of the PennPET Explorer using analytic (3DFRP) and iterative (LM-TOF-OSEM, 5 iterations) reconstruction algorithms. Radial, tangential, and axial resolutions (FWHM) are reported at radial positions (r) of 1 and 20 cm, averaged over 3 axial positions.

*r* (cm)	Algorithm	Radial (mm)	Tangential (mm)	Axial (mm)
1	analytic	4.1 ± 0.3	4.2 ± 0.1	4.0 ± 0.2
1	iterative	3.9 ± 0.3	3.8 ± 0.3	3.6 ± 0.2
20	analytic	5.7 ± 0.2	5.2 ± 0.3	4.8 ± 0.1
20	iterative	5.6 ± 0.3	4.0 ± 0.5	3.4 ± 0.1

#### Image quality (IQ)

The NEMA image quality (IQ) phantom with standard-sized spheres (10, 13, 17, 22, 28 and 37 mm in diameter) was filled and imaged following the NEMA NU 2-2018 protocol. All spheres were filled with ^18^F at a contrast of 3.92 with respect to the background, which had an activity concentration of 5.4 kBq cc^−1^. Two 20 × 30 cm uniform phantoms matching the background activity concentration of the IQ phantom were placed on each end of the IQ phantom. The IQ phantom was scanned for 30 min on the 6-ring scanner without axial gaps at two axial positions—center of AFOV and off-center between the first two rings—to evaluate how the variations in axial resolution and sensitivity affect the image quality.

Data were reconstructed using LM-TOF-OSEM (2 × 2 × 2 mm^3^ voxels), and the contrast recovery coefficient (CRC) and background variability (BV) were calculated from reconstructed images following NEMA NU 2-2018. While the NEMA protocol dictates a 30 min scan, this is much longer than typical FDG scans on TB-PET systems; therefore, additional analyses were performed on 3 min scans (which were generated by subsampling the 30 min-duration list-mode data into 10 replicates).

#### Clinical Trials Network phantom

The Clinical Trials Network (CTN) phantom is not part of NEMA NU-2 standard but is used in a variety of SNM-sponsored clinical trials that include long AFOV systems. The phantom is larger and more anthropomorphic than the IQ phantom and includes smaller (7 mm) spheres. It contains 12 spheres with diameters ranging from 7 to 37 mm, with a 7 mm sphere included in the uniform background and 5 spheres (including two 10 mm ones virtually contiguous to each other) in two asymmetric lung fields. The phantom was used to assess performance for two different radionuclides: ^18^F and ^89^Zr and to evaluate how the system performs under the challenges associated with ^89^Zr studies (i.e. low injected dose, low positron fraction, high energy non-prompt gammas in the decay scheme that increase the measured randoms). The CTN phantom was filled with ^18^F at a background activity concentration of 6.67 kBq cc^−1^ with lesion contrast of 4.17:1; for the ^89^Zr study the background activity concentration was 1.25 kBq cc^−1^ (i.e. 5.3x lower than for ^18^F) with a lesion contrast of 4.95:1. The activity concentrations were selected to mimic those seen in the clinic, where tissue activity concentrations of ∼5 kBq cc^−1^ (^18^F) and ∼0.5 kBq cc^−1^ (^89^Zr) are obtained; the lesion contrasts were set to be similar to that of IQ phantom. The phantom was scanned at the center of AFOV for 60 min (^89^Zr) and 11.3 min (^18^F) to achieve equal activity-scan duration (75 kBq min cc^−1^) for the two studies to isolate the impact of the lower positron fraction with ^89^Zr. Image reconstruction and calculation of CRC and BV followed the same methodology as for NEMA IQ phantom, except that only 10 mm ROIs were used to determine the BV for the CTN phantom.

## Results

### Sensitivity

Table 2 shows the sensitivity results for different configurations of the PennPET Explorer. The measured total sensitivity was 140.2 kcps MBq^−1^ with a 70 cm line source at the center of AFOV for the 6-ring PennPET Explorer with all detectors enabled. The measured sensitivity gain for the scanner in its current configuration (without gaps) is ∼1.8x compared to the scanner configuration with axial gaps (i.e. 5-row readout), which closely agrees with the geometric estimation on the gain in sensitivity of 1.9x. This increase in sensitivity further extends the imaging capabilities of the system and potentially enables the use of lower injected doses.

**Table 2.** Total sensitivity at the center of the FOV for the 6-ring PennPET Explorer (with and without axial gaps) and for the 1-ring-segment scanner without axial gaps. Published results for the 5-ring PennPET Explorer (Viswanath* et al* 2020) are also included for reference.

# rings	Gap	AFOV (cm)	70 cm Sens (kcps/MBq)	Peak sens (kcps/(MBq/cm))	70 cm Sens (kcps/(MBq/cm))	142 cm Sens (kcps/(MBq/cm))
6	N	142	140	30	9800	14430
6	Y	136	77^a^	21	5390	7920
5	Y	112	69^a^	19	4830	6360
1	N	22.9	8	7.9	560	n/a

^a^ 70 cm sensitivity extrapolated from 142 cm measurement.

**Figure 3.**
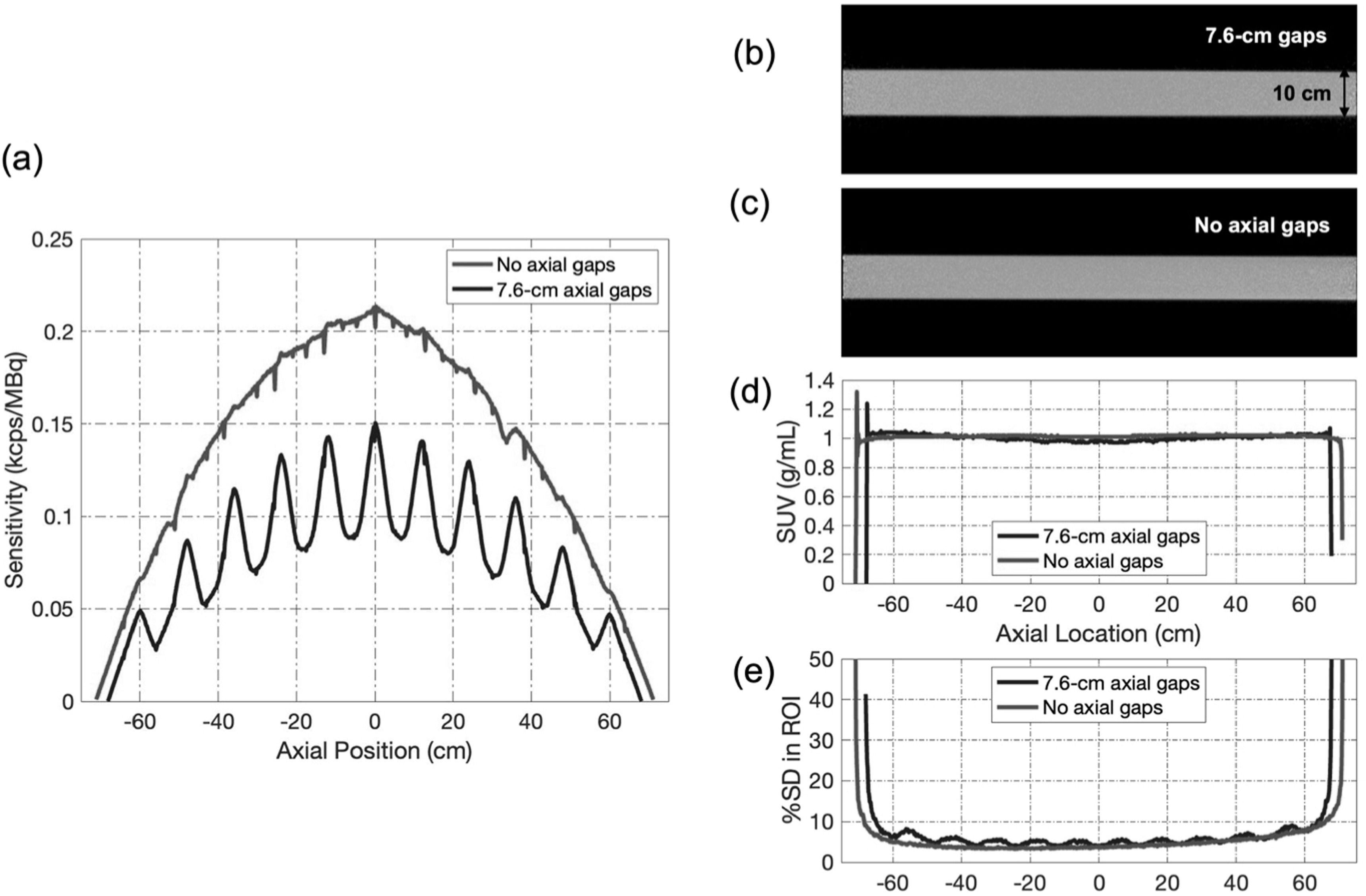
(a) Axial sensitivity profiles measured with a 142 cm line source on the 6-ring PennPET Explorer with and without axial gaps. 2 mm coronal image slice of the reconstructed 10 × 190 cm pipe phantom uniformly filled with 120 MBq (8.4 kBq cc^−1^) of ^18^F and imaged in a single bed position for 15 min on the 6-ring scanner (b) with and (c) without axial gaps. (d) Axial uniformity (SUV) and (e) axial noise (image roughness) profile of the reconstructed pipe phantoms.

Figure 3(a) shows the measured axial sensitivity profiles for the 6-ring scanner with and without gaps. The peak sensitivity increases by ∼40% with the 7-row readout firmware. The shape of the axial sensitivity profile is roughly triangular, as with all 3D systems with unrestricted acceptance angle; the slight deviation from a triangular profile is due to geometrical effects with the large axial acceptance angle. Measurements at the off-center position (*r* = 10 cm), not shown, were consistent with the measurements at *r* = 0.

### Image uniformity

The variation in the axial sensitivity profile with the 7.6 cm axial gaps (figure 3(a)) is caused by the varying number of lines of response (LORs) with axial position due to inactive detectors with the 5-row readout firmware. The non-uniformity of axial sensitivity with axial gaps is compensated in the reconstruction with normalization correction (Karp *et al* 2020), as can be seen in the images of the long pipe (figure 3(b)). The SUVs of the pipe phantom range from 0.97 to 1.03 except for the extreme edges (figure 3(d)), indicating excellent uniformity across the AFOV with and without axial gaps. While there is some variation in the noise behavior due to the axial gaps seen in figure 3(e), this is not visible in the images (compare figures 3(b) and (c)). 

### Count rate and accuracy

**Figure 4.**
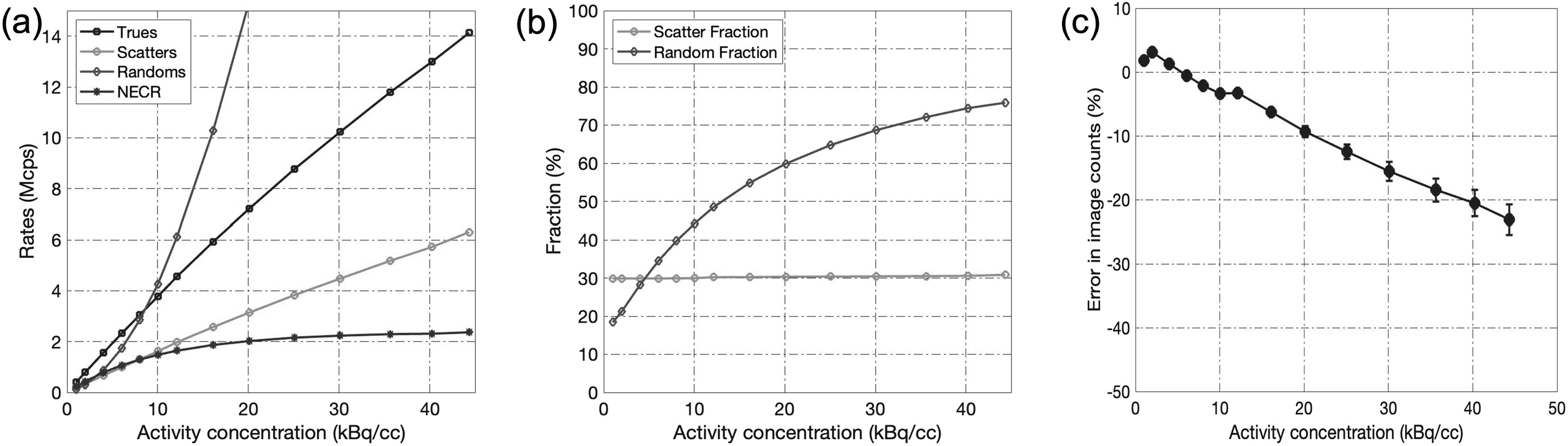
Count rate performance measured with the 20 × 70 cm NEMA phantom on the PennPET Explorer in its current configuration, 6 rings without axial gaps. (a) Trues, scatter, randoms and noise-equivalent count (NEC) rates, (b) the corresponding scatter fraction and randoms fraction, and (c) accuracy as a function of activity concentration.

The count rate performance measured with a 20 × 70 cm NEMA phantom for the 6-ring scanner in without axial gaps is shown in figure 4. The trues rate is almost linear with activity (figure 4(a)), with small (<5%) deadtime below 10 kBq cc^−1^, as seen in figure 4(c). The scatter fraction is stable (29.9%–30.8%) over a wide range of activities up to 45 kBq cc^−1^, indicating stable energy peaks (i.e. no light pile-up) owing to the 1:1 crystal-detector coupling in the detector design. This observation is consistent with 3-ring (Karp *et al* 2020) and 5-ring (Viswanath *et al* 2020) results due to the modular design of the system—adding more rings does not add additional deadtime to the system. A peak NEC rate was not measured; the NEC rate continues to increase with activity and reaches 2360 kcps at 44 kBq cc^−1^, the maximum activity concentration measured. The NEC rate is ∼1.3 Mcps when the randoms and trues rates are equal at ∼8 kBq cc^−1^.

**Figure 5.**
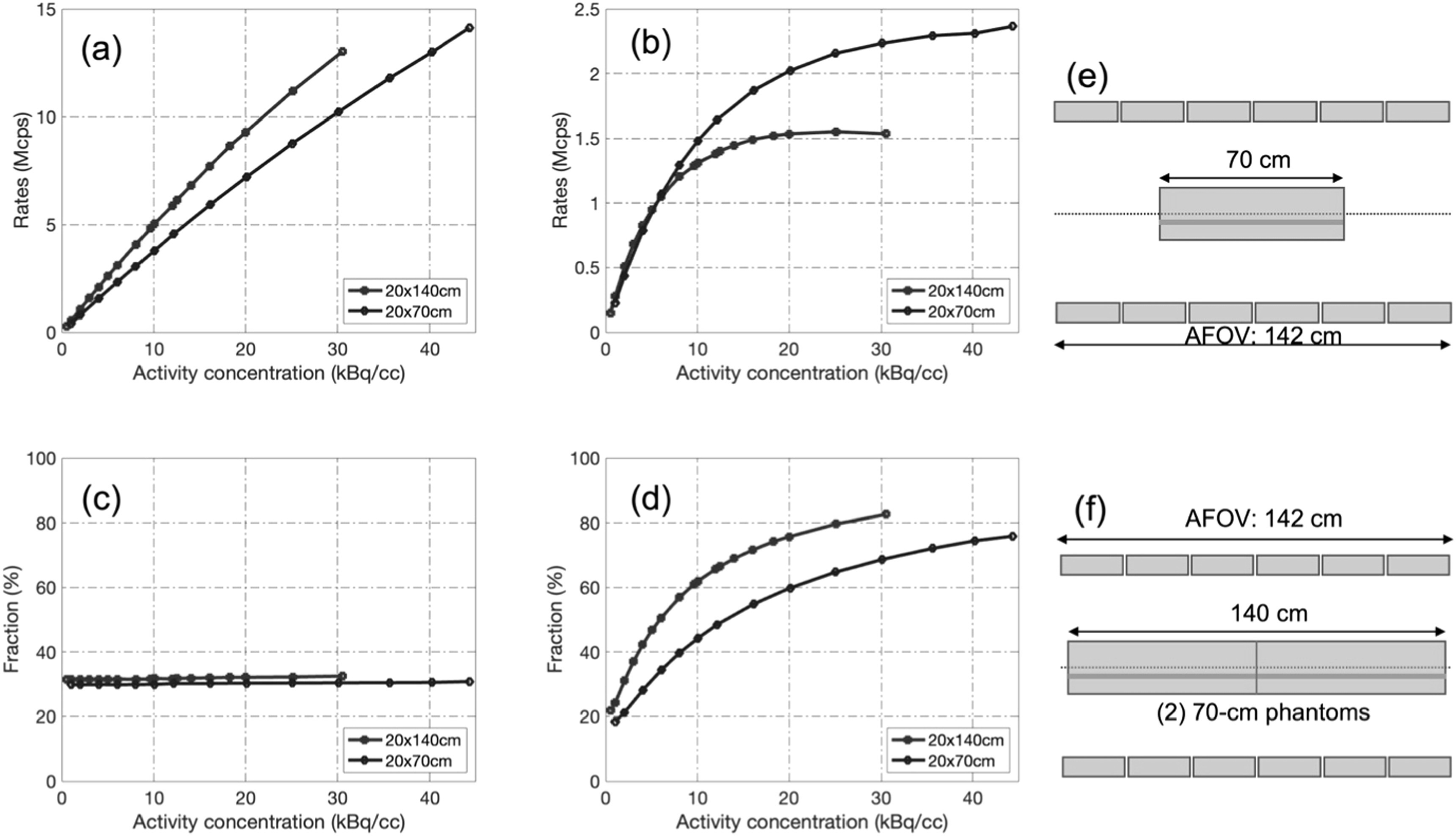
Count rate performance measured with single 20 × 70 cm and double (20 × 140 cm) NEMA phantoms on the PennPET Explorer without axial gaps. (a) Trues rates, (b) NEC rates, (c) scatter fractions, and (d) randoms fractions as a function of activity concentration. Schematic depicting the placement of (e) the 20 × 70 cm and (f) the 20 × 140 cm count rate phantoms.

Figure 5 shows the comparison of the count rate performance of the 6-ring scanner measured with the single NEMA 20 × 70 cm and double 20 × 140 cm phantoms. The trues rate (figure 5(a)) is higher with the 140 cm phantom due to the increased number of LORs, as is the randoms fraction (figure 5(d)). The scatter fraction (figure 5(c)) with the 140 cm phantom is ∼32% over a wide range of activities, only a small increase compared to that measured with the 70 cm phantom. The NEC rates (figure 5(b)) are similar for the two phantoms at lower activities (<8 kBq cc^−1^) but are lower with the longer phantom at higher activities due to the increased number of randoms. However, most human studies are performed with activity concentrations <8 kBq cc^−1^. The corresponding NEC rate is ∼0.75-1 Mcps in the clinical range of activities.

**Figure 6.**
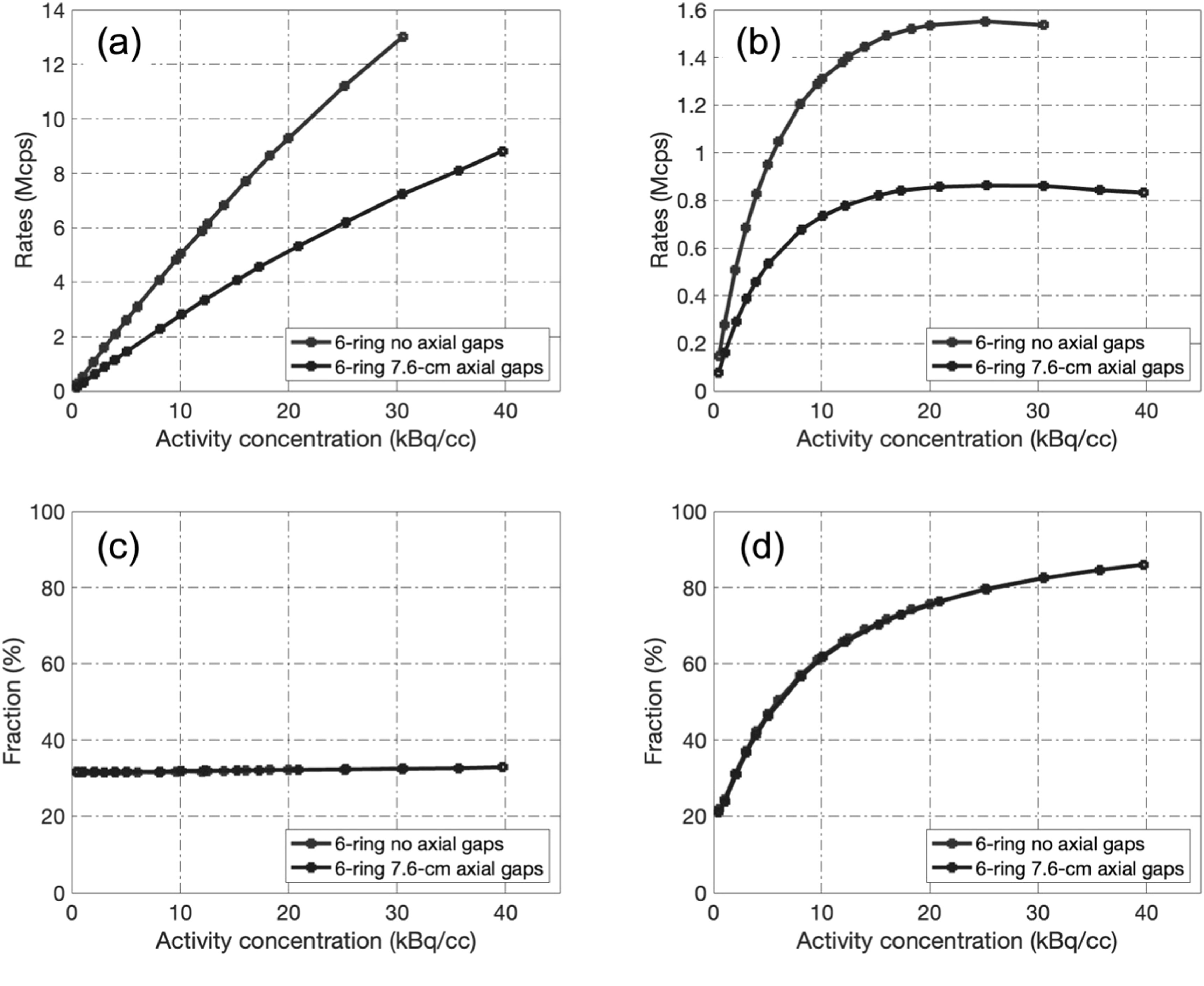
Count rate performance measured with the double (20 × 140 cm) NEMA phantom on the PennPET Explorer with and without axial gaps. (a) Trues rates, (b) NEC rates, (c) scatter fractions, and (d) randoms fractions as a function of activity concentration.

Figure 6 shows the comparison of the count rate performance of the 6-ring scanner with and without gaps measured with the double 20 × 140 cm phantom. The peak NEC rates are 860 kcps and 1550 kcps with and without axial gaps respectively, both at 25 kBq cc^−1^ with the same scatter fraction of 32%. Both the trues rate and NEC rate increased by 1.8x without the axial gaps compared to the configuration with gaps across the entire range of activity concentrations. This agrees well with the 1.8x increase in measured sensitivity that results from the inclusion of 40% more detectors within the fixed axial FOV.

### TOF resolution

**Figure 7.**
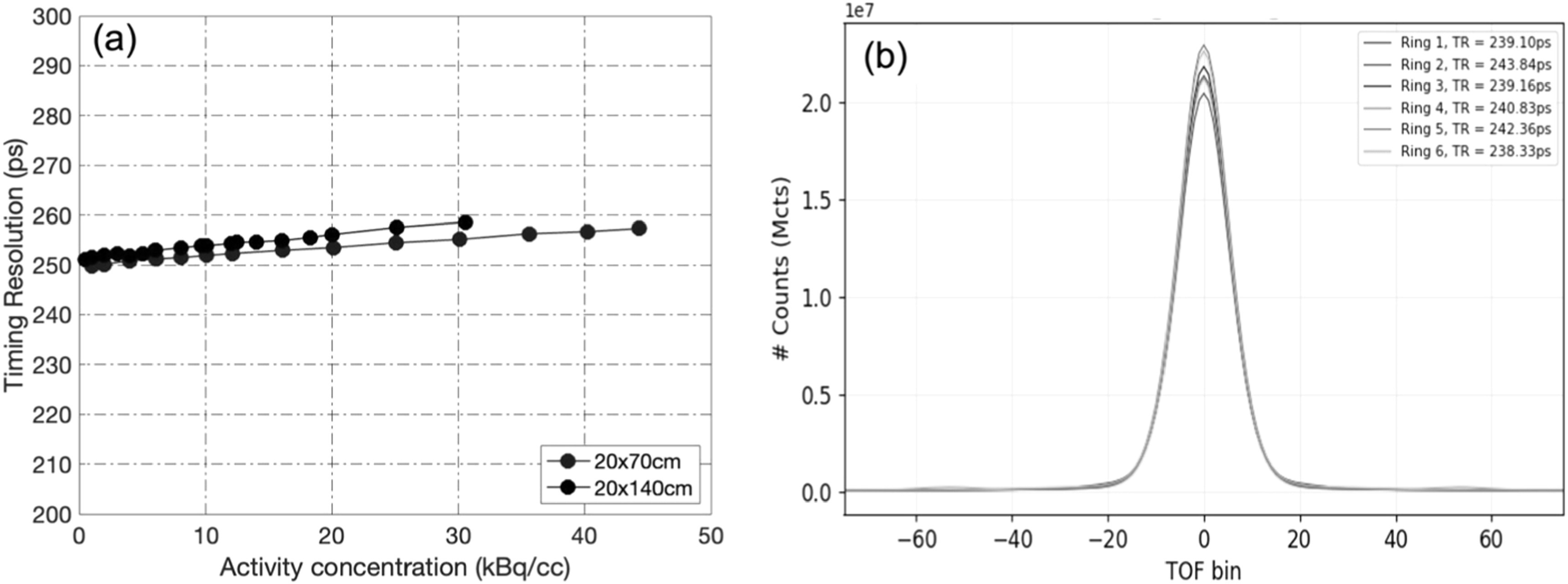
(a) Time-of-flight resolution measured with single 20 × 70 cm and double (20 × 140 cm) NEMA phantoms on the PennPET Explorer without axial gaps. (b) Timing histograms of single rings from daily QC measurements performed with a point source in the center of each ring. The timing resolution per ring averages 240 ps.

The timing resolution of the 6-ring scanner is stable and the difference is minimal (<3 ps) between 70- and 140 cm phantoms over a wide range of activities (figure 7(a)); in addition, the degradation with increasing activity is small: 251 ps at 5 kBq cc^−1^ and 256 ps at 30 kBq cc^−1^. The timing resolution measured with the NEMA count-rate phantom, which includes a measure of all LORs (compared to the narrow axial acceptance with one ring), is only ∼10 ps higher at low activity than the average single-ring timing resolution of 240 ps from daily QC (figure 7(b)), indicating very good timing calibration and synchronization of data from all six rings.

### Spatial resolution

**Figure 8.**
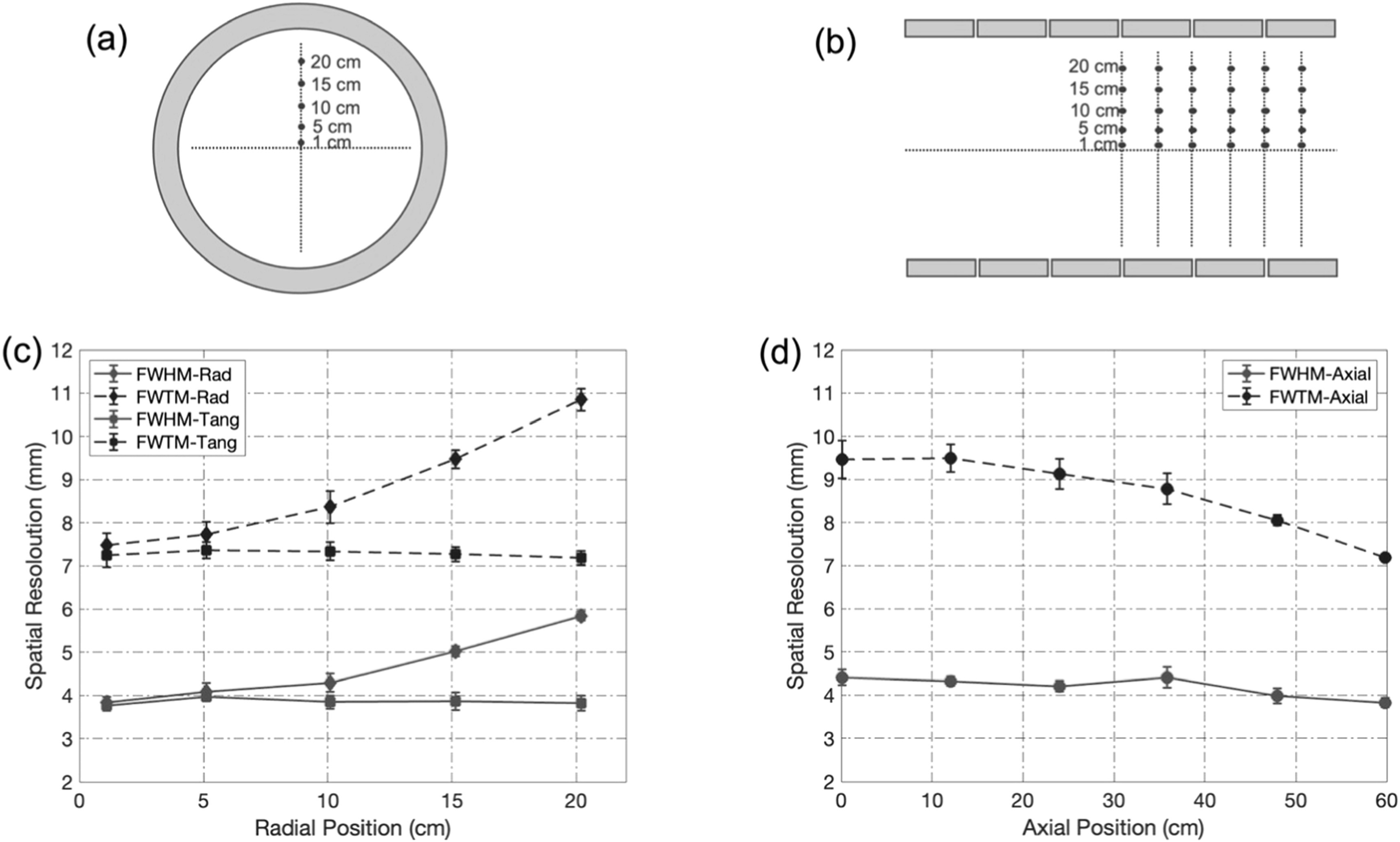
Spatial resolution performance measured on the PennPET Explorer with 6 rings without axial gaps. Schematic depicting (a) the transversal and (b) the axial locations of the measurements (c) Radial and tangential resolution as a function of radial position. (d) Axial resolution as a function of axial position.

The spatial resolution of the 6-ring scanner is shown in figure 8. Tangential resolution does not change from the center of the transverse FOV to the radial edge. Radial resolution degrades by 2 mm for FWHM and by 4 mm for FWTM at a radial position of 20 cm compared with the radial center, as expected for a cylindrical system due to parallax errors. Axial FWHM degrades from 3.8 mm at the end of the scanner to 4.4 mm in the center of AFOV because the center position corresponds to a larger acceptance angle (±62°), which leads to more depth-of-interaction uncertainties for oblique LORs. Nevertheless, the degradation of only 0.6 mm even with such a large acceptance angle is smaller than that seen in the radial direction. The measured results agree well with prior simulations (Daube-Witherspoon *et al* 2021).

### Image quality (IQ)

**Figure 9.**
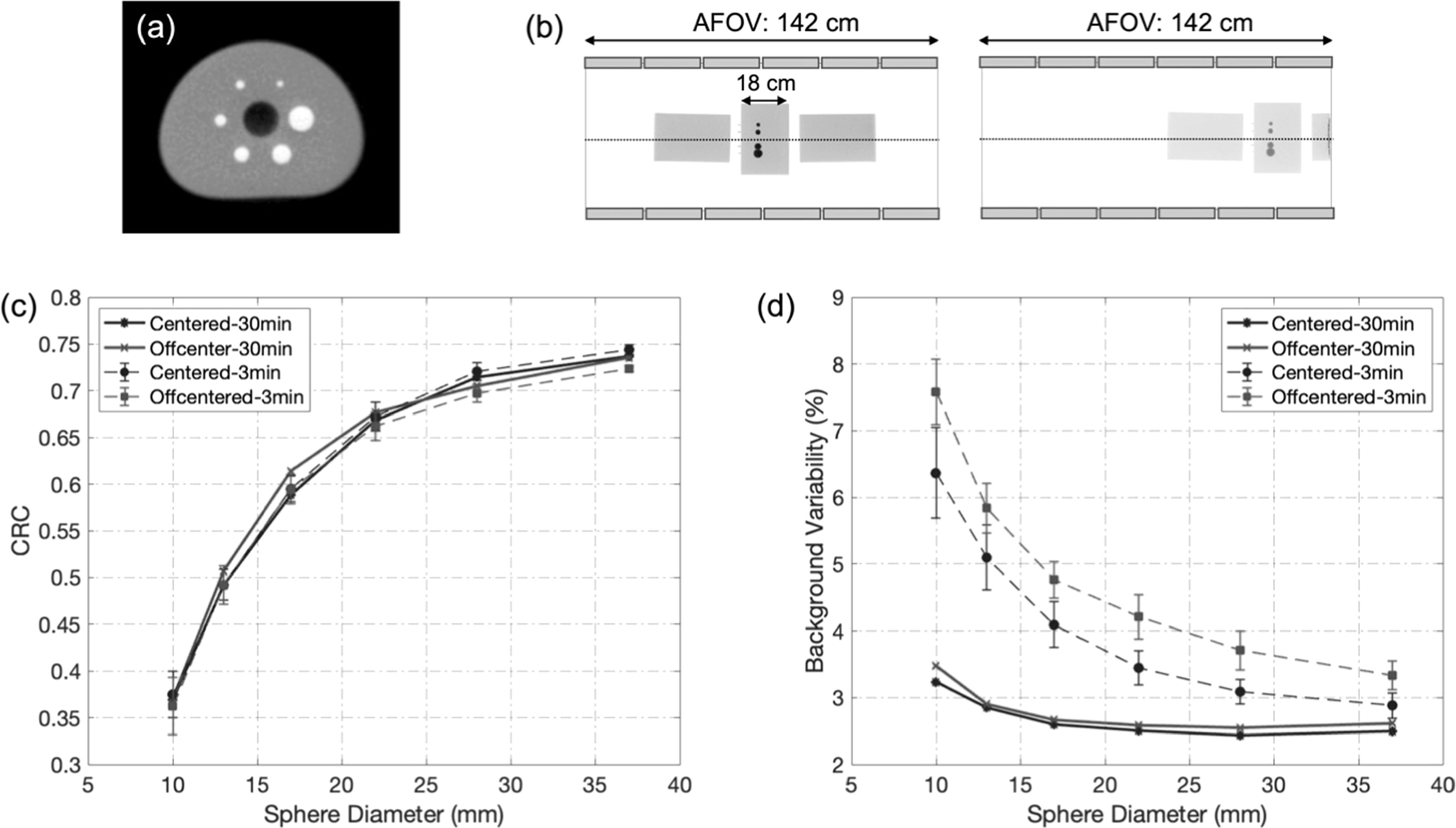
(a) Transaxial image slice (2 mm thick) of the standard NEMA image quality phantom placed at the center of the AFOV and imaged for 30 min on the PennPET Explorer without axial gaps. (b) Schematic of 6 detector rings overlaid with maximum-intensity projection (MIP) of the IQ phantom depicting the center and off-center axial locations where the phantom was imaged. (c) Contrast recovery coefficient (CRC) and (d) background variability measured at two axial positions for 30 min and 3 min scans on the 6-ring system without axial gaps.

The NEMA IQ phantom was measured at two axial positions—center of AFOV, between rings 3 and 4, and off-center (1/6 AFOV) between rings 1 and 2—for both 30 min and 3 min scans, to assess how the variations in axial resolution, sensitivity, and scan duration affect the image quality of the long-AFOV system. As shown in figure 9(c), the CRC is not affected by scan duration, as expected. More importantly, the CRC is similar for the center and off-center positions, for all sphere sizes, demonstrating that the small loss of axial resolution at the center of the AFOV does not impact lesion quantitative accuracy for lesions as small as 10 mm. Figure 9(d) shows that the noise (BV) is lower with longer scans, as expected, and somewhat higher for the off-center position for the 3 min scans due to the reduced sensitivity at the off-center position compared with the center. Overall, consistent lesion quantification is demonstrated across the entire AFOV.

### Clinical Trials Network phantom

**Figure 10.**
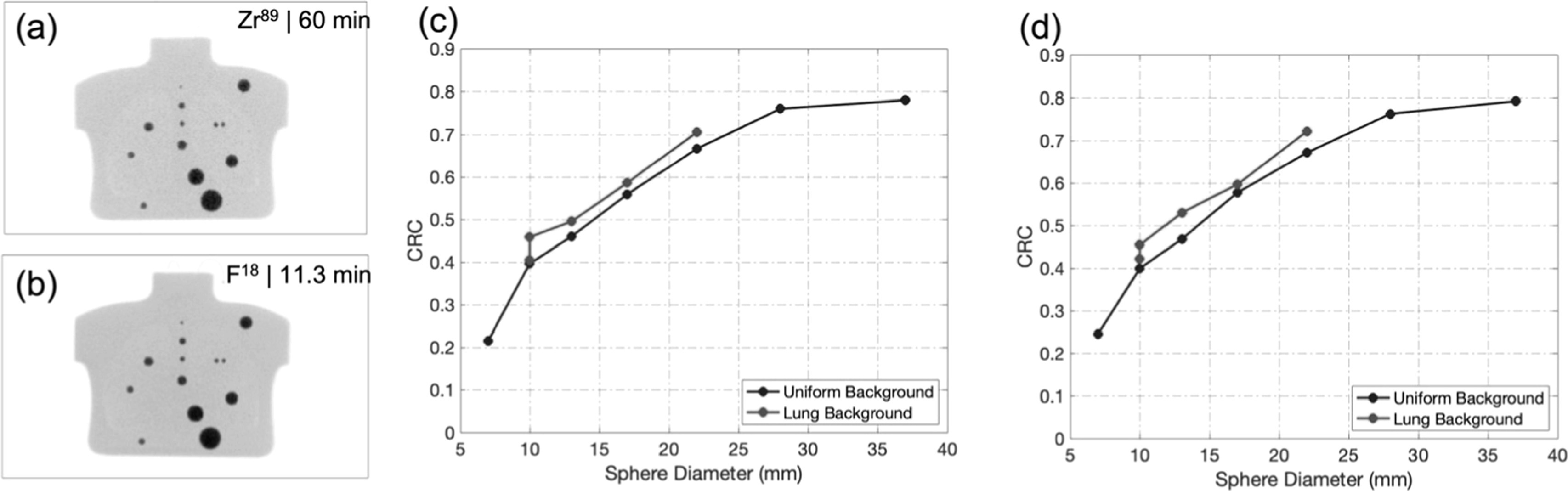
Maximum intensity projection (MIP) images of the Clinical Trials Network (CTN) phantom filled with (a) ^89^Zr and (b) ^18^F. The phantoms were placed at the center of AFOV and scanned for 60 min and 11.3 min respectively for equal activity-scan duration. Contrast recovery measured with the (c) ^89^Zr and (d) ^18^F CTN phantom on the 6-ring PennPET Explorer without axial gaps.

The CTN phantom was measured with both ^18^F and ^89^Zr to comply with requirements of a clinical trial and to complement the NEMA IQ measurements that are only performed with ^18^F. At equal activity-scan durations, the trues rate of the CTN phantom filled with ^89^Zr is ∼25% of that with ^18^F due to the reduced positron fraction with ^89^Zr (0.223, versus 0.969 for ^18^F). The 4x difference in counts contributes to 1.3x difference in BV (4.5% for ^89^Zr-CTN versus 3.5% for ^18^F-CTN in the uniform background), considering that image noise is not strictly statistical due to image reconstruction, and the BV metric is not directly a measure of statistics. The MIP image of the ^89^Zr-CTN (figure 10(a)) has comparable visual quality as that with ^18^F (figure 10(b)), and the CRCs are similar for both cases (figures 10(c), (d)). Overall, the comparison of the results with ^89^Zr and ^18^F demonstrates that ^89^Zr can be imaged with low noise and good lesion quantitative accuracy on a long AFOV system, despite the challenges associated with ^89^Zr imaging, i.e. low injected dose (typically 37 MBq), low positron fraction, and high positron energy.

## Discussion

### Firmware

The upgrade of the PennPET Explorer to 142 cm AFOV to eliminate the inter-ring axial gaps was made possible owing to the modular, scalable, and programmable design of the system, in particular the FPGA-based data acquisition firmware. The new 7-row readout firmware was designed in a way that any detector can be independently enabled/disabled from the acquisition software. Therefore, it not only can dynamically adjust the hardware resources for data acquisition for a particular study, but it also opens new opportunities in exploring cost-effective scanner designs on hardware with incomplete detector coverage. The firmware can also be reconfigured (within the FPGA resource) to improve or expand the capability of the system, e.g. implementing new event positioning and timing algorithms.

### Sensitivity

The firmware upgrade increases detector coverage by 40% and leads to a 1.8x increase in sensitivity for both 70 cm and 142 cm line sources, as expected. The sensitivity is 140 kcps/MBq for NEMA 70 cm line source. However, we recognize that the measured sensitivity, although significantly higher than on a conventional scanner, is lower than expected for a TB-PET system with 142 cm AFOV and unrestricted acceptance of LORs. We are aware of two factors that reduce our sensitivity compared to predictions based on geometry and preliminary results with 1 to 3 rings. First, the 1.1 cm gaps between rings (equivalent to 4.2% of the length of a single ring) and non-functioning detector tiles (3.3% of total tiles) on the scanner together lead to 15% loss in sensitivity compared to a geometry with 100% coverage. Many of the non-functioning tiles were ignored before the firmware update to read all 7 rows and in principle can be replaced during a scheduled shutdown. The detector functionality is monitored during daily QC and very few tiles fail during operation, so reliability is expected to remain high. Second, as the system was expanded beyond 3 rings we have chosen to raise the nominal temperature of the scanner from 5˚ to 10˚C to maximize reliability and minimize the possibility of condensation on the electronics; however, the sensitivity decreases as temperature increases, due to the influence of dark noise on deadtime for the digital SiPM-based detector tiles. We have measured a 17% drop in sensitivity from 5 °C to 10 °C while operating with trigger 1 in the lab. Since the detector tiles are very reliable, i.e. the majority of the failures did not occur during use, there are no current plans or urgency to replace the non-functioning tiles nor lower the temperature, despite the modest impact on sensitivity.

As shown in figure 1, the clinical activity distribution can extend beyond 70 cm, and the wide variation of sensitivity across the AFOV seen in figure 3 demonstrates the need to fully capture this variation across the entire TB-PET system, something that cannot be done with a 70 cm long source. We used a longer line (>= AFOV) to capture the full axial sensitivity profile. If one assumes that the activity in a patient is distributed along the AFOV, a longer AFOV system will capture more events by imaging more of the body. One way to capture this increase in sensitivity is to scale the sensitivity not by the total activity in the line but by the *activity/cm*, so that results from measurements for systems with different AFOVs and line source lengths can be fairly compared. The metrics of interest therefore become the *total sensitivity per activity/cm* and the *peak sensitivity per activity/cm*. Doing this for the PennPET Explorer with 6 rings results in a 47% gain in total sensitivity for the 142 cm line compared with the 70 cm line, with and without axial gaps (table 2). The peak sensitivity increases more modestly as AFOV increases, consistent with the calculations shown in figure 1(b) of (Daube-Witherspoon *et al* 2022). Although we did not have difficulty filling the longer line source uniformly, the sensitivity measurement may be simplified if performed with moving a point source through the AFOV.

### Count rate

The NEC rate with the NEMA (single) 70 cm phantom achieves 2360 kcps at the maximum activity concentration that we measured (44 kBq cc^−1^), even though most human and large animal imaging occurs below 8 kBq cc^−1^. The system deadtime is low, <5% up to 10 kBq cc^−1^; thus, we did not implement deadtime corrections in this work or our prior work (Karp *et al* 2020), although we plan to do so in the future to compensate for the deadtime at higher activities. Timing resolution is 251 ps and stable with count rate (<2 ps increase at 10 kBq cc^−1^). Scatter fraction is 30% and stable with count rate. The 1:1 crystal-to-detector coupling (thus, small deadtime and no light pile-up) plays a crucial role in the consistently good count rate performance. Measurements with a longer phantom demonstrate a higher randoms fraction that is closer to what is seen for human imaging (50%–75% for activity concentration 5–10 kBq cc^−1^) and therefore suggests that a phantom longer than 70 cm be considered for long AFOV systems to better approximate the noise equivalent count rate (NECR) for clinical imaging.

### Spatial resolution

Using our default iterative reconstruction algorithm, the spatial resolution at the edge of the AFOV is very close to the result of 4 mm previously obtained using analytic reconstruction for a single ring (see table 1), with about ∼0.6 mm (2 mm) FWHM (FWTM) degradation near the center of the AFOV with an unrestricted axial acceptance angle. Some concerns (Berg *et al* 2016, Vandenberghe *et al* 2020, Wang *et al* 2022) were raised that the axial parallax error introduced by long AFOV (with a large axial acceptance angle) can significantly degrade the spatial resolution and the quantitative accuracy of small structures, which will offset the sensitivity gain of TB PET scanners. However, our measurement showed that such impact on quantitative recovery of small structures appears minimal (see figure 9(c)), as we also observed in simulation studies of long AFOV systems (Daube-Witherspoon *et al* 2021). In addition, a separate measurement with a point source embedded in a 20 cm diameter warm cylinder (not shown) agrees well with our measurements of a point source in air when using our iterative algorithm with the same reconstruction parameters; the transverse spatial resolution changed negligibly, while the axial spatial resolution improved slightly due to preferential attenuation of the oblique coincidence pairs.

Note that with the iterative reconstruction the radial resolution worsens as radial location increases, while the tangential and axial resolutions remain constant with radial position (table 1), as expected. We recognize that using an iterative reconstruction for point sources in air is difficult to standardize. Our implementation of LM-TOF-OSEM with spherical image basis functions, however, does not show the artificial enhancement of resolution with more iterations (i.e. the results for 20 iterations are equal to those for the standard 5 iterations, not shown). Therefore, we believe these results fairly characterize the PSF of the PennPET Explorer. We suggest that the NEMA standard for imaging spatial resolution should be changed to be less sensitive to rebinning or sampling errors not encountered in clinical imaging and to better reflect performance seen in patient imaging that is routinely performed with iterative reconstruction approaches. Several proposals have been made that may be practical: to use a line source, arranged diagonally to allow for measures in axial and transverse directions, within a water phantom and report spatial resolution versus background noise, such as proposed by Kinahan *et al* (2016), or more simply basing the measurement on the edge of a water filled cylindrical phantom positioned obliquely (Lodge *et al* 2018).

### Image quality

Although not belonging to the NEMA standard, a long pipe phantom was used to demonstrate excellent axial uniformity, an important metric for a long AFOV system. This measurement was particularly important to demonstrate uniform axial uniformity while the system was in its interim configuration with inter-ring axial gaps because these caused local variations in the axial sensitivity beyond the normal center-edge variation present with all 3D systems. Lesion uptake measurements with NEMA IQ phantom demonstrate the need to revise the scan time requirement (30 min to scan 1 m) for long AFOV systems, since high CRC and low BV are achieved on the PennPET Explorer even with 3 min scans. The CTN phantom, more anthropomorphic than the NEMA IQ, demonstrates similar performance as the NEMA IQ, and was used also to show correspondence for ^18^F and ^89^Zr scans, an important consideration for enlisting a long AFOV scanner in clinical trials. ^89^Zr scans have similar CRC for spheres between 10 and 37 mm, but higher BV due to its low positron fraction.

Finally, we note that this work is not intended as a complete NEMA performance evaluation but rather to characterize the performance of the PennPET Explorer (as it relates to imaging performance for large animals and humans) and to illustrate the challenges associated with using the current NEMA standards for long AFOV systems. As such, the PET-CT alignment measurement was not included, as the technique is similar to that for standard PET/CT systems.

## Conclusion

The 6-ring-segment PennPET Explorer has been completed with updated data acquisition firmware to achieve 142 cm AFOV without inter-ring axial gaps. The design of the TB-PET system was described, and the modification to the readout firmware to enable all detectors was explained. NEMA NU 2-2018 metrics were extended to quantify the system. The performance evaluation of the PennPET Explorer shows that detector gaps may provide a solution for more cost-effective TB-PET, with the trade-off of a factor of 1.8x lower sensitivity and NECR for a system with 40% fewer detectors for comparable axial coverage.

## Data Availability

The data that support the findings of this study are openly available at the following URL/DOI: https://doi.org/10.7910/DVN/RVNXQQ. Data will be available from 13 March 2023.

## Ethical statement

The research was approved by the Institutional Review Board (IRB) at the Perelman School of Medicine (PSOM) at the University of Pennsylvania School of Medicine, and was conducted in accordance with the principles embodied in the Declaration of Helsinki and in accordance with local statutory requirements. All research subjects are with informed consent under IRB # 843546.

## References

[pmbacc722bib1] Badawi Ramsey D, Karp Joel S, Nardo Lorenzo, Pantel Austin R (2021). Total Body PET Imaging: Exploring New Horizons. PET Clinics.

[pmbacc722bib2] Berg E, Roncali E, Kapusta M, Du J, Cherry S R (2016). A combined time-of-flight and depth-of-interaction detector for
total-body positron emission tomography. Med. Phys..

[pmbacc722bib3] Daube-Witherspoon Margaret E, Pantel Austin R, Pryma Daniel A, Karp Joel S (2022). Total-body PET: a new paradigm for molecular imaging. The British Journal of Radiology.

[pmbacc722bib4] Daube-Witherspoon M E, Viswanath V, Werner M E, Karp J S (2021). Performance characteristics of long axial field-of-View PET scanners
with axial gaps. IEEE Trans. Radiat. Plasma Med. Sci..

[pmbacc722bib5] Degenhardt C, Prescher G, Frach T, Thon A, de Gruyter R, Schmitz A, Ballizany R (2009). The digital silicon photomultiplier–a novel sensor for the detection
of scintillation light.

[pmbacc722bib6] Degenhardt C, Rodrigues P, Trindade A, Zwaans B, Mulhens O, Dorscheid R, Thon A, Salomon A, Frach T (2012). Performance evaluation of a prototype positron emission tomography
scanner using digital photon counters (DPC).

[pmbacc722bib7] Frach T, Prescher G, Degenhardt C, de Gruyter R, Schmitz A, Ballizany R (2009). The digital silicon photomultiplier - Principle of operation and
intrinsic detector performance.

[pmbacc722bib8] Gong K, Cherry S R, Qi J (2016). On the assessment of spatial resolution of PET systems with iterative
image reconstruction. Phys. Med. Biol..

[pmbacc722bib9] Karp J S, Viswanath V, Geagan M J, Muehllehner G, Pantel A R, Parma M J, Perkins A E, Schmall J P, Werner M E, Daube-Witherspoon M E (2020). PennPET Explorer: design and preliminary performance of a whole-body
imager. J. Nucl. Med..

[pmbacc722bib10] Kinahan P (2016). Task-oriented quantitative performance assessments for comparing
PET/CT systems. J. Nucl. Med..

[pmbacc722bib11] Lodge M A, Leal J P, Rahmim A, Sunderland J J, Frey E C (2018). Measuring PET Spatial resolution using a cylinder phantom positioned
at an oblique angle. J. Nucl. Med..

[pmbacc722bib12] Matej S, Lewitt R M (1996). Practical considerations for 3D image reconstruction using spherically
symmetric volume elements. IEEE Trans. Med. Imaging.

[pmbacc722bib13] Matej S, Lewitt R M (2001). 3D-FRP: Direct Fourier reconstruction with Fourier reprojection for
fully 3D PET. IEEE Trans. Nucl. Sci..

[pmbacc722bib14] Matej S, Surti S, Jayanthi S, Daube-Witherspoon M E, Lewitt R M, Karp J S (2009). Efficient 3D TOF PET reconstruction using view-grouped histo-images:
DIRECT-direct image reconstruction for TOF. IEEE Trans. Med. Imaging.

[pmbacc722bib15] Miller M, Zhang J, Binzel K, Griesmer J, Laurence T, Narayanan M, Natarajamani D, Wang S, Knopp M (2015). Characterization of the vereos digital photon counting PET
system. J. Nucl. Med..

[pmbacc722bib16] (2018). NEMA Standards Publication NU 2-2018 Performance Measurements of Positron
Emission Tomographs (PET)..

[pmbacc722bib17] Pantel A R, Viswanath V, Daube-Witherspoon M E, Dubroff J G, Muehllehner G, Parma M J, Pryma D A, Schubert E K, Mankoff D A, Karp J S (2020). PennPET explorer: human imaging on a whole-body imager. J. Nucl. Med..

[pmbacc722bib18] Popescu L M, Matej S, Lewitt R M (2004). Iterative image reconstruction using geometrically ordered subsets
with list-mode data.

[pmbacc722bib19] Prenosil G A, Sari H, Fürstner M, Afshar-Oromieh A, Shi K, Rominger A, Hentschel M (2022). Performance characteristics of the biograph vision quadra PET/CT
system with long axial field of view using the NEMA NU 2-2018
standard. J. Nucl. Med..

[pmbacc722bib20] Rausch I, Ruiz A, Valverde-Pascual I, Cal-González J, Beyer T, Carrio I (2019). Performance evaluation of the vereos PET/CT system according to the
NEMA NU2-2012 standard. J. Nucl. Med..

[pmbacc722bib21] Spencer B A (2021). Performance evaluation of the uexplorer total-body PET/CT scanner
based on NEMA NU 2-2018 with additional tests to characterize PET scanners with a
long axial field of view. J. Nucl. Med..

[pmbacc722bib22] Vandenberghe S, Moskal P, Karp J S (2020). State of the art in total body PET. EJNMMI Physics.

[pmbacc722bib23] Viswanath V, Daube-Witherspoon M E, Pantel A R, Parma M J, Werner M E, Karp J S (2020). Performance benefits of extending the AFOV of PET
scanners.

[pmbacc722bib24] Wang Z (2022). High-resolution and high-sensitivity PET for quantitative molecular
imaging of the monoaminergic nuclei: a GATE simulation study. Med. Phys..

[pmbacc722bib25] Werner M E, Surti S, Karp J S (2006). Implementation and evaluation of a 3D PET single scatter simulation
with TOF modeling.

[pmbacc722bib26] Yamaya T, Yoshida E, Inadama N, Nishikido F, Shibuya K, Higuchi M, Murayama H (2009). A multiplex ‘openPET’ geometry to extend axial FOV without increasing
the number of detectors. IEEE Trans. Nucl. Sci..

[pmbacc722bib27] Zein S A, Karakatsanis N A, Conti M, Nehmeh S A (2021). Monte carlo simulation of the siemens biograph vision PET with
extended axial field of view using sparse detector module rings
configuration. IEEE Trans. Rad. Plasma Med. Sci..

